# Small-Pox

**Published:** 1847-04

**Authors:** 


*Small-Pox.*—M. Béhier, one of the physicians of *La Charité*, Paris, has had during the last few weeks several interesting cases of small-pox in his service. A female, twenty-eight years of age, slept for some nights next to the bed of a patient labouring under variola: one morning, in making up a bed, she touched his person. Twenty days after she was seized with chilliness, followed by heat and a sensation of general lassitude, with pains in the loins, felt particularly in movements of the limbs. She complained of soreness of the throat, with tolerably intense oppression; the tongue was white, the eyes weeping, and the palate the seat of papulæ. The most remarkable phenomenon in this case, and it is on this account that I have reported it, was the form and volume of the pustules, which were small, pointed, resembling simple acne, and not presenting the appearance of pustules with wide bases which are observed in variola. This modification in the eruption alone gave rise to some fears of unpleasant complications. And this is a character which it is well to bear in mind, that whenever the pustules present themselves in this way, there is reason to anticipate an abnormal march, or some grave epiphenomenon in the disease. The third day a violent delirium declared itself; the patient jumped out of bed and ran across the ward. She complained greatly of her throat: her pulse was at first 74; then 84; then 90. Six leeches were applied behind each ear. Calomel was given internally; two large blisters to the lower extremities, after having previously used sinapisms. The third day after the delirium the pustules became less distinct, and the symptoms improved. This is a remarkable fact—the patient had been vaccinated, and should have had varioloid; but, on the contrary, her symptoms were exceedingly grave. It is generally the case, when a patient who has been vaccinated is attacked with variola, that the disease is more mild than if vaccination had not been performed. An opinion prevails very extensively among physicians, that in variola the pustule is umbilical, or pointed, and that it is not so in varioloid. This is proved to be altogether erroneous. In variola, umbilical pustules are but rarely seen, and the only real difference between it and varioloid is in the time of desiccation. In the former, between the eruption and the desiccation, there is a distinct period, while in varioloid the pustules appear, become dry, and the scales are formed and fall off very rapidly.

There should be no importance attached to the umbilical shape of the pustules in these diseases, according to M. Béhier, who has made extensive and careful researches on the subject, both at *l'Hospital Saint Louis* and *l'Hospital des Enfans*, where he has had numerous opportunities of observing these diseases. As a general rule, variola does not offer umbilical pustules. The history of this patient, after her recovery from variola, shows the possibility of complications posterior to the principal affection. The parotid of the left side of her neck inflamed, and the abscess was opened. Erysipelas seized first this side, and afterwards the other, and was followed by anasarca, which commenced in the face, obstinate diarrhœa, and albuminous urine. The same remedy was employed in this condition, as in a similar case supervening upon an attack of scarlatina: vapour baths produced sensible amelioration of the symptoms.—*Ibid.*

